# Association between viscoelastic tests-guided therapy with synthetic factor concentrates and allogenic blood transfusion in liver transplantation: a before-after study

**DOI:** 10.1186/s12871-018-0664-8

**Published:** 2018-12-22

**Authors:** Raffael P. C. Zamper, Thiago C. Amorim, Veronica N. F. Queiroz, Jordana D. O. Lira, Luiz Guilherme V. Costa, Flavio Takaoka, Nicole P. Juffermans, Ary S. Neto

**Affiliations:** 1 Department of Transplant Anesthesia, Hospital Israelita Albert Einstein, Rua Galeno de Almeida 107 ap 172-A, Pinheiros, SP 05410-030 Brazil; 2 Resident of the Anesthesiology Program, Hospital Israelita Albert Einstein, São Paulo, Brazil; 3 Department of Critical Care Medicine, Hospital Israelita Albert Einstein, São Paulo, Brazil; 4 Department of Intensive Care, Academic Medical Center, University of Amsterdam, Amsterdam, The Netherlands

**Keywords:** Liver transplantation, Blood coagulation disorders, Blood transfusion, Hemostasis, Hemorrhage, Prothrombin complex concentrate, Fibrinogen

## Abstract

**Background:**

Perioperative bleeding and transfusion are important causes of morbidity and mortality in patients undergoing liver transplantation. The aim of this study is to assess whether viscoelastic tests-guided therapy with the use of synthetic factor concentrates impact transfusion rates of hemocomponents in adult patients undergoing liver transplantation.

**Methods:**

This is an interventional before-after comparative study. Patients undergoing liver transplantation before the implementation of a protocol using thromboelastometry and synthetic factor concentrates were compared to patients after the implementation. Primary outcome was transfusion of any hemocomponents. Secondary outcomes included: transfusion of red blood cells (RBC), fresh frozen plasma (FFP), cryoprecipitate or platelets, clinical complications, length of stay and in-hospital mortality.

**Results:**

A total of 183 patients were included in the control and 54 in the intervention phase. After propensity score matching, the proportion of patients receiving any transfusion of hemocomponents was lower in the intervention phase (37.0 vs 58.4%; OR, 0.42; 95% CI, 0.20–0.87; *p* = 0.019). Patients in the intervention phase received less RBC (30.2 vs 52.5%; OR, 0.21; 95% CI, 0.08–0.56; *p* = 0.002) and FFP (5.7 vs 27.3%; OR, 0.11; 95% CI, 0.03–0.43; *p* = 0.002). There was no difference regarding transfusion of cryoprecipitate and platelets, complications related to the procedure, hospital length of stay and mortality.

**Conclusions:**

Use of a viscoelastic test-guided transfusion algorithm with the use of synthetic factor concentrates reduces the transfusion rates of allogenic blood in patients submitted to liver transplantation.

**Trial registration:**

This trial was registered retrospectively on November 15th, 2018 – clinicaltrials.gov – Identifier: NCT03756948.

**Electronic supplementary material:**

The online version of this article (10.1186/s12871-018-0664-8) contains supplementary material, which is available to authorized users.

## Introduction

Perioperative bleeding is one of the most important causes of morbidity and mortality in liver transplantation [1]. However, blood transfusion, used to correct hemorrhage and coagulopathy, is directly associated with an increase in infectious and respiratory complications [2, 3], longer intensive care unit (ICU) length of stay, and a higher rate of reoperations [4–6], increasing mortality among these patients [7]. In addition, transfusion of packed red blood cells (RBC) was shown to be associated with the incidence of hepatic artery thrombosis [8] and the use of cryoprecipitate, platelets and fresh frozen plasma (FFP) associated with decreased graft survival at one and five years [9]. The decision to transfuse a patient undergoing liver transplantation presents as a challenge, and more than a half of patients undergoing liver transplantation still require transfusion of some blood product components in the perioperative period [9–15].

Patients with advanced liver diseases present with changes in coagulation and hemostasis, including an elevated international normalized ratio (INR), decreased levels of fibrinogen and a decreased platelet count, and these abnormal values suggest a state of hypocoagulability [16]. However, thrombin generating capacity is normal or even increased in this group of patients when compared to healthy controls [17, 18], and the platelets are qualitatively capable of withstanding adequate thrombin generation when their total count is around 50–60 × 10^9^/l [19]. Other features of a hypercoagulable profile include increased von Willebrand factor levels, high amounts of procoagulant platelet-derived microparticles and a hypofibrinolytic state [20]. At the end, hemostasis finds a new and fragile equilibrium [16] and the isolated conventional laboratory tests are inefficient to evaluate the coagulation status [21, 22].

Thromboelastography (TEG®, Haemoscope / Haemonetics, Niles, Ill) as a method to assess global hemostatic function through a simple blood sample was described in 1948 and has been used in liver transplantation since the 1980s [23, 24]. Rotational thromboelastometry (ROTEM®) adopts the same principles of TEG, as a method that assess the viscoelastic property of whole blood allowing the evaluation of the initiation, formation, stability and lysis of the clot [23]. These point-of-care (POC) tests have become complementary tools to traditional static tests [25, 26], and recent studies have shown that coagulation assessment and viscoelastic tests-guided therapy during high risk procedures, such as cardiovascular surgery and trauma, can have a significant impact on the reduction of transfusion of blood products and also in the morbidity and mortality of the patients [27, 28].

Some studies support the use of viscoelastic tests (VET) in the management of perioperative liver transplant coagulation [29–31], adding valuable real-time information during the different stages of surgery. However, strategies based on these tests are still under development and the best triggers for blood transfusion are not completely known. Prior to 2007, patients in Brazil were transplanted in order of waiting list, regardless of disease severity, causing patients to undergo liver transplantation at very different stages of the disease [32], with subsequent lower transfusion rates during procedure [33]. The adoption of the ‘Model for End-Stage Liver Disease’ (MELD) as an organ allocation method in places with low offers of organs for donation has changed this practice. Although currently evidence suggest benefit of the use of VET in this group of patients, the impact of this intervention in patients undergoing liver transplantation in Brazil according to the MELD system is not known.

The aim of the present before-after study is to assess whether VET-guided therapy with the use of synthetic factor concentrates (fibrinogen concentrate [FC] and prothrombin complex concentrate [PCC]) is associated with decreased transfusion of blood product components in adult patients undergoing liver transplantation in a private hospital in Brazil using MELD as an organ allocation method.

## Methods

### Ethics statement

The protocol was approved by the local ethics committee of Hospital Israelita Albert Einstein (Comitê de Ética do Hospital Israelita Albert Einstein, São Paulo, Brazil). Written consent was applied to patients in the prospective group (intervention group), and was waived in the retrospective group (control group).

### Patients and setting

The present study was performed in the operating room and in the ICU of a private teaching hospital. Data from adult patients undergoing liver transplantation were collected and analyzed. All patients undergoing deceased donor liver transplantation for chronic liver disease were considered for inclusion, and in our center split organs and donation after circulatory death are not used. The following exclusion criteria were considered: transplantation due to acute liver failure, age < 18 years old, combined transplant recipients (e.g., liver and kidney) and those who require re-transplantation in less than thirty days after the first transplant.

### General Care for Liver Transplantation

Patients were admitted to the operating room without receiving any pre-anesthetic medication, and were monitored with electrocardiogram, pulse oximetry and bi-spectral index (BIS). A 16-gauge venous access and a radial arterial line were acquired before anesthetic induction. After intubation, a central venous access was obtained in jugular vein preferably, and all patients were monitored with transesophageal echocardiography (TEE).

In a specific group of patients, a pulmonary-artery catheter was also used (presence of pulmonary hypertension, cardiomyopathy, or a MELD score above 30). The type of anesthesia (total intravenous or balanced) was left at the discretion of the anesthesiologist. During the whole period the same groups of anesthesiologists and surgeons were responsible for all the liver transplantations. All patients used elastic stockings and an intermittent pneumatic compressor in the lower limbs, and prophylactic heparin was not allowed during the hospitalization according to institutional protocol. At the end, all patients were referred to the ICU.

### Study protocol

#### Study design

A before-after study design was used. The before period (control phase) consisted of patients undergoing liver transplantation who were operated between 2007 and 2009, at least five years before the implementation of a protocol using VET and synthetic factor concentrates. We opted for patients operated during this period to avoid a bias, because before 2009 we did not have neither synthetic factor concentrates for using in these patients nor POC-VET available in our center.

The intervention phase consisted of patients undergoing liver transplantation operated during a 10-month period after this implementation (January 2015 to October 2015). Although this strategy led to a gap of at least five years between control and intervention cases, the team of surgeons and anesthesiologists remained the same, both with more than five years of experience in liver transplantation.

#### Control phase

In the control phase, transfusion of RBC was triggered by either a hemoglobin (Hb) value below 7.0 g/dL or by signs of hemodynamic instability (persistent hypotension, tachycardia, low arterial oxygen content, severe and acute bleeding with hypotension). Strategy of transfusion and choice of blood product to treat coagulopathies was performed guided by previous laboratory results of conventional coagulation tests when there was clinical evidence of coagulopathy, active bleeding and normal metabolic profile (pH, temperature and serum calcium). Synthetic factor concentrates were available, but we did not have institutional authorization for using them in an off-label setting, so the patients in the control phase did not receive these concentrates.

Antifibrinolytics were used prophylactically in all cases when there was no history of inflammatory diseases from the biliary tract, hepatocellular carcinoma and no previous thromboembolic event. There was no fluid administration protocol, but in our practice, we use albumin combined with crystalloids and tend to be restrictive with fluids administration, using the four chambers view on TEE to estimate if the heart is empty, hyperdynamic or dysfunctional. This evaluation associated with assessment of the mean arterial pressure (MAP) and central venous pressure (CVP) supported our decisions on fluid resuscitation.

ROTEM® was not available during this period. We performed a retrospective analysis of prospectively recorded data regarding demographic characteristics, laboratory tests, medications, surgical characteristics, strategy of fluid replacement, use of blood products (blood product components), vital signs, general complications after surgery, including thromboembolic complications (myocardial infarction, stroke, deep venous thrombosis, pulmonary thromboembolism or portal thrombosis), postoperative duration of mechanical ventilation, ICU and hospital length of stay, and mortality.

#### Intervention phase

During a 10-month period, the recommended procedure was to treat coagulopathies according to a strategy of transfusion based on the results of VET and to use synthetic factor concentrates instead of hemocomponents. As in the control phase, transfusion of RBC was triggered by either a Hb value under 7,0 g/L or by signs of hemodynamic instability and fluid resuscitation was exactly as in the control group. Patients were followed until hospital discharge or death, whichever came first.

**Fig. 1:**
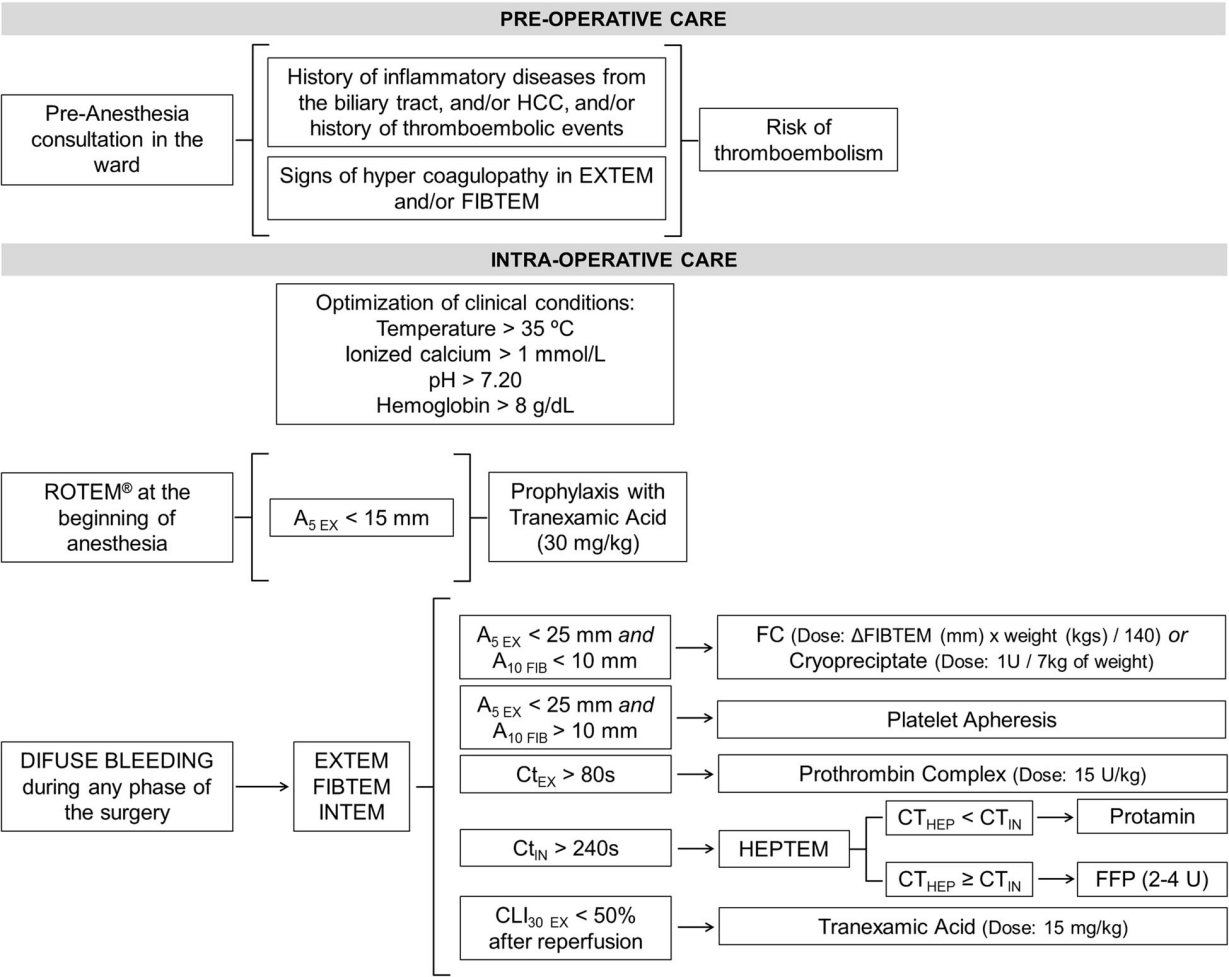
Algorithm for assessment and treatment of alteration of coagulation during liver transplantation. *HCC: hepatocellular carcinoma; ROTEM: rotational thromboelastometry; A*_*5 EX*_*: clot firmness after 5 min in EXTEM; A*_*10 EX*_*: clot firmness after 10 min in FIBTEM; Ct*_*EX*_*: clotting time in EXTEM; CT*_*IN*_*: clotting time in INTEM; CT*_*HEP*_*: clotting time in HEPTEM; CLI*_*30 EX*_*: clot lysis index after 30 min in EXTEM; FFP: fresh frozen plasma; FC: fibrinogen concentrate*

Coagulation treatment was indicated when there was a clinical coagulopathy with bleeding, and management was based on a POC-VET algorithm adapted from those used in cardiovascular surgeries, designed in conjunction with hematologists and experts in the area and using an earlier amplitude evaluation in the EXTEM at the fifth minute (A5_EX_) (Fig. 1) [34]. Antifibrinolytics were indicated when there was no history of inflammatory diseases from the biliary tract, hepatocellular carcinoma, no previous thromboembolic event, and if the A5_EX_ amplitude was under 15 mm in the ROTEM® performed in the beginning of the anesthesia.

ROTEM® was performed in the following moments: in the preoperative period within the routine laboratory exams in the ward, 15 min after arterial reperfusion, six hours after the end of the transplant in the ICU and at any time when the team considered necessary based on clinical aspects of the surgical field.

### Outcomes

The primary outcome was a collapsed composite of need of any transfusion of blood product during surgery and in the first 48 h in the postoperative, and this included the need of RBC, FFP, cryoprecipitate and/or platelets. Secondary outcomes were: 1) use of synthetic factor concentrates or antifibrinolytic; 2) clinical complications related to the procedure; 3) postoperative duration of ventilation in days; 4) ICU and hospital length of stay in days; and 5) in-hospital mortality.

### Statistical analysis

The control phase has been set to liver transplantations performed between 2007 and 2009 and the intervention phase 10-months duration a priori. The control to intervention ratio was set as 3:1. The primary analysis consisted in comparing any transfusion of blood product components between the two phases by means of a chi-square test. To consider potential bias associated with the before-after design, we performed two analyses with an adjustment for demographic characteristics. First, a multivariate analysis was performed using a logistic or a linear regression model including variables differing between the two phases in bivariate analyses and those already know as prognostic factors for transfusion in liver transplantation. Variables used for adjustment were age, MELD score, Child-Pugh classification, presence of hepatocellular carcinoma (HCC), and preoperative levels of albumin, urea, creatinine and hemoglobin. Second, a propensity score method was applied to balance covariates in the two phases and to reduce bias. Propensity scores were estimated for each patient with logistic regression using age, Child-Pugh, MELD, presence of HCC and preoperative levels of hemoglobin as co-variates. The propensity score reflects the propensity in the range of 0 to 1 to be in the intervention phase given a set of known variables and is an attempt to adjust for potential selection bias, confounding factors, and differences between groups. Patients with missing data were excluded from the analysis. Based on the propensity score weighted estimators for the data we constructed a propensity score–matched cohort. Matching was performed using nearest neighbor matching without replacement, with each patient from the intervention phase matched to two patients of the control phase. A caliper width of 0.1 of the standard deviation of the logit of the propensity score was used for the development of matching.

A logistic or a linear regression was performed on this matched sample. All results are presented as odds ratio (OR) and it 95% confidence interval (95% CI) for logistic regression or the β coefficient and it 95% CI for linear regression.

Normality of the variables was tested with a Kolmogorov-Smirnoff test. Continuous parametric data were expressed as the mean (SD), and nonparametric data were expressed as median and interquartile range. Categorical data were expressed as absolute numbers and percentage. For demographic characteristics Student t test was used as appropriate. All analyses were conducted with SPSS v.20 (IBM SPSS Statistics for Windows, Version 20.0. Armonk, NY: IBM Corp.), and R v.2.12.0. For all analyses two–sided *p* values < 0.05 were considered significant.

## Results

### Population

Demographic characteristics of included population are reported in Table 1. One hundred and eighty-three patients were included in the control and fifty-four in the intervention phase. At baseline, patients in the intervention phase had lower incidence of chronic kidney disease, encephalopathy and upper digestive hemorrhage, lower preoperative levels of creatinine and urea and higher levels of albumin (Table 1). However, there were no differences between the groups when comparing MELD scores. The characteristics of the groups were more balanced after the propensity-score matching (Table 1).

**Table 1:** Baseline characteristics of the patients

	Unmatched Cohort (*n* = 237)			Matched Cohort (*n* = 135)^b^		
	Intervention (*n* = 54)	Control (*n* = 183)	*p* value^a^	Intervention (*n* = 46)	Control (*n* = 89)	*p* value^a^
Baseline characteristics and co-morbidities						
Age, years	53.0 ± 11.1	51.9 ± 12.0	0.550	53.0 ± 11.8	52.5 ± 11.9	0.791
Gender, male	43 / 54 (79.6)	131 / 183 (71.6)	0.239	35 / 46 (76.1)	71 / 89 (79.8)	0.620
Weight, kg	77.8 ± 15.9	76.0 ± 17.0	0.483	76.2 ± 16.5	76.7 ± 15.7	0.867
Height, cm	171.2 ± 8.0	168.3 ± 9.9	0.051	170.2 ± 7.8	170.1 ± 8.6	0.927
BMI, kg/m^2^	26.6 ± 4.4	26.7 ± 5.1	0.848	26.3 ± 4.6	26.4 ± 4.6	0.945
Co-morbidities						
Chronic kidney disease	3 / 54 (5.6)	34 / 183 (18.6)	0.020	2 / 46 (4.3)	17 / 89 (19.1)	0.019
Hypertension	14 / 54 (25.9)	31 / 183 (16.9)	0.139	13 / 46 (28.3)	14 / 89 (15.7)	0.084
Diabetes mellitus	11 / 54 (20.4)	48 / 183 (26.2)	0.381	9 / 46 (19.6)	28 / 89 (31.5)	0.141
Etiology of liver disease			0.578			0.852
Budd-Chiari	0 / 54 (0.0)	3 / 182 (1.6)		0 / 46 (0.0)	1 / 89 (1.1)	
Alcohol	14 / 54 (25.9)	32 / 182 (17.6)		11 / 46 (23.9)	22 / 89 (24.7)	
Hepatitis C	23 / 54 (42.6)	89 / 182 (48.9)		19 / 46 (41.3)	40 / 89 (44.9)	
Hepatitis B	2 / 54 (3.7)	13 / 182 (7.1)		2 / 46 (4.3)	8 / 89 (9.0)	
Cryptogenic	5 / 54 (9.3)	16 / 182 (8.8)		4 / 46 (8.7)	6 / 89 (6.7)	
PSC	2 / 54 (3.7)	6 / 182 (3.3)		2 / 46 (4.3)	2 / 89 (2.2)	
Autoimmune hepatitis	2 / 54 (3.7)	6 / 182 (3.3)		2 / 46 (4.3)	3 / 89 (3.4)	
FAP	0 / 54 (0.0)	5 / 182 (2.7)		0 / 46 (0.0)	0 / 89 (0.0)	
Acute Liver Failure	0 / 54 (0.0)	2 / 182 (1.1)		0 / 46 (0.0)	1 / 89 (1.1)	
Others	6 / 54 (11.1)	10 / 182 (5.5)		6 / 46 (13.0)	6 / 89 (6.7)	
Clinical status pre-transplantation						
Use of mechanical ventilation	2 / 54 (3.7)	14 / 183 (7.7)	0.309	2 / 46 (4.3)	6 / 89 (6.7)	0.576
Use of hemodialysis	4 / 54 (7.4)	16 / 183 (8.7)	0.756	3 / 46 (6.5)	7 / 89 (7.9)	0.777
Previous surgery	8 / 54 (14.8)	37 / 183 (20.2)	0.373	8 / 46 (17.4)	14 / 89 (15.7)	0.804
Child-Pugh classification			0.019			0.223
A	13 / 54 (24.1)	44 / 183 (24.0)		13 / 46 (28.3)	17 / 89 (19.1)	
B	7 / 54 (13.0)	57 / 183 (31.1)		7 / 46 (15.2)	24 / 89 (27.0)	
C	34 / 54 (63.0)	82 / 183 (44.8)		26 / 46 (56.5)	48 / 89 (53.9)	
MELD score	22.7 ± 8.8	21.6 ± 8.2	0.418	21.9 ± 9.2	22.0 ± 7.9	0.954
Re-transplantation	1 / 54 (1.9)	0 / 183 (0.0)	0.065	1 / 46 (2.2)	0 / 89 (0.0)	0.162
Complications						
Encephalopathy	1 / 54 (1.9)	94 / 183 (51.4)	< 0.001	1 / 46 (2.2)	50 / 89 (56.2)	< 0.001
Upper digestive hemorrhage	1 / 54 (1.9)	67 / 183 (36.6)	< 0.001	1 / 46 (2.2)	37 / 89 (41.6)	< 0.001
Portal vein thrombosis	4 / 54 (7.4)	10 / 183 (5.5)	0.594	3 / 46 (6.5)	4 / 89 (4.5)	0.614
Portopulmonary hypertension	1 / 54 (1.9)	4 / 183 (2.2)	0.880	1 / 46 (2.2)	2 / 89 (2.2)	0.978
HCC	23 / 54 (42.6)	53 / 183 (29.0)	0.059	16 / 46 (34.8)	28 / 89 (31.5)	0.696
Ascites			0.082			
Controlled	13 / 54 (24.1)	37 / 183 (20.2)		12 / 46 (26.1)	26 / 89 (29.2)	0.083
Refractory	14 / 54 (25.9)	78 / 183 (42.6)		11 / 46 (23.9)	35 / 89 (39.3)	
Medications in use						
Furosemide	22 / 54 (40.7)	53 / 183 (29.0)	0.101	20 / 46 (43.5)	27 / 89 (30.3)	0.128
Spironolactone	27 / 54 (50.0)	68 / 183 (37.2)	0.013	23 / 46 (50.0)	37 / 89 (41.6)	0.350
Propranolol	27 / 54 (50.0)	58 / 183 (31.7)	0.090	22 / 46 (47.8)	33 / 89 (37.1)	0.228
Pre-transplantation laboratory tests						
INR	1.8 ± 0.8	1.7 ± 0.6	0.113	1.8 ± 0.8	1.7 ± 0.7	0.532
Total bilirubin, mg/dL	5.4 ± 8.0	6.2 ± 8.1	0.532	4.8 ± 7.6	7.2 ± 9.6	0.149
Albumin, g/dL	3.2 ± 0.5	3.0 ± 0.6	0.025	3.3 ± 0.5	3.0 ± 0.6	0.004
Hemoglobin, g/dL	11.3 ± 2.5	11.3 ± 2.6	0.641	11.3 ± 2.7	10.9 ± 2.5	0.433
Hematocrit, %	32.5 ± 7.0	32.5 ± 7.5	0.954	32.6 ± 7.4	31.9 ± 7.4	0.633
Fibrinogen, mg/dL	214.5 ± 92.6	178.3 ± 94.5	0.301	214.5 ± 92.6	153.7 ± 70.2	0.095
Platelets, × 1000/mm_3_	80.5 ± 59.2	74.2 ± 55.0	0.473	81.6 ± 62.7	67.1 ± 48.7	0.143
Urea, mg/dL	36.5 ± 15.7	49.7 ± 43.3	0.030	35.9 ± 14.9	51.9 ± 47.8	0.028
Creatinine, mg/dL	0.9 ± 0.4	1.2 ± 0.8	0.004	0.9 ± 0.4	1.2 ± 0.7	0.008
Sodium, mEq/L	137.1 ± 3.3	137.5 ± 5.8	0.702	137.3 ± 3.0	137.4 ± 6.1	0.963
Potassium, mEq/L	4.3 ± 0.5	4.1 ± 0.5	0.074	4.3 ± 0.5	4.2 ± 0.5	0.316
Ejection fraction of LV, %	66.2 ± 8.5	67.0 ± 7.3	0.577	66.4 ± 8.9	68.3 ± 6.6	0.253

*Kg: kilograms; cm: centimeters; BMI: body mass index; PSC: Primary Sclerosing Cholangitis; MELD: Model for End-Stage Liver Disease; FAP: Familial Amyloid Polyneuropathy; INR: international normalized ratio; mg: miligrams; dL; deciliters; g: grams; HCC: hepatocellular carcinoma; LV: left ventricle*

Data presented as mean ± standard deviation or number / total (percentage)

^a^Comparison of differences between the two groups using the *t* test for continuous variables and the χ^2^ test for categorical variables

^b^Adjusted by age, Child, MELD, presence of HCC and pre-transplantation hemoglobin

### Postoperative and surgical characteristics

The clamping and ischemia time was lower in the intervention phase compared to the control phase (Table 2). Intraoperatively, patients in the intervention phase received less fluid and had lower fluid balance than patients in the control phase. At the end of the surgery, patients in the intervention phase presented with a lower heart rate, CVP, and temperature and higher MAP and dose of norepinephrine compared to patients in the control phase (Table 2).

**Table 2:** Postoperative and surgical characteristics

	Unmatched Cohort (*n* = 237)			Matched Cohort (*n* = 135)^b^		
	Intervention (*n* = 54)	Control (*n* = 183)	*p* value^a^	Intervention (*n* = 46)	Control (*n* = 89)	*p* value^a^
General characteristics of the surgery						
Technique			0.586			0.978
Piggy-back	53 / 54 (98.1)	177 / 183 (96.7)		45 / 46 (97.8)	87 / 89 (97.8)	
Conventional	1 / 54 (1.9)	6 / 183 (3.3)		1 / 46 (2.2)	2 / 89 (2.2)	
Clamping time, minutes	38.1 ± 10.3	64.8 ± 90.0	0.032	38.3 ± 10.6	52.4 ± 17.9	< 0.001
Ischemia time, minutes	418.3 ± 69.2	569.9 ± 165.8	< 0.001	421.0 ± 71.8	584.4 ± 161.3	< 0.001
Use of fluids, urine output and cell-saver						
Total fluid infusion, mL	3846.3 ± 1124.01	4986.7 ± 1811.3	< 0.001	3743.5 ± 956.7	4946.9 ± 1734.6	< 0.001
Lactated ringer, mL	0.0 ± 0.0	710.1 ± 1524.2	0.001	0.0 ± 0.0	398.9 ± 991.9	0.007
Normal saline, mL	0.0 ± 0.0	118.8 ± 510.7	0.089	0.0 ± 0.0	134.8 ± 542.3	0.095
Plasma-Lyte®, mL	3570.4 ± 1055.7	3733.1 ± 2171.5	0.595	3484.8 ± 925.9	4000.0 ± 2012.7	0.102
Albumin, mL	275.9 ± 134.9	424.7 ± 317.5	0.001	258.7 ± 118.1	413.2 ± 173.5	< 0.001
Cell-saver, mL	522.4 ± 713.6	568.0 ± 968.0	0.748	585.0 ± 734.8	653.6 ± 1066.8	0.697
Urine output, mL	611.3 ± 510.1	722.1 ± 431.4	0.117	644.4 ± 529.7	722.1 ± 441.8	0.372
Fluid balance, mL	3248.1 ± 1212.0	4254.3 ± 1737.6	< 0.001	3112.2 ± 1021.5	4237.2 ± 1689.9	< 0.001
Vital signs and vasopressors						
Induction						
Heart rate, bpm	75.3 ± 13.9	78.9 ± 14.9	0.117	76.1 ± 13.1	79.6 ± 15.1	0.188
Systolic pressure, mmHg	118.7 ± 26.9	117.8 ± 20.9	0.808	120.2 ± 26.8	118.5 ± 19.0	0.674
Diastolic pressure, mmHg	62.5 ± 14.5	61.3 ± 13.1	0.546	63.8 ± 14.3	61.0 ± 13.4	0.258
Mean arterial pressure, mmHg	80.7 ± 17.7	80.3 ± 16.2	0.877	81.4 ± 17.8	79.8 ± 15.3	0.569
Central venous pressure, mmHg	10.2 ± 3.7	12.8 ± 4.0	< 0.001	10.1 ± 3.9	12.9 ± 3.9	0.001
Temperature, °C	36.1 ± 0.4	36.2 ± 0.6	0.574	36.2 ± 0.4	36.1 ± 0.6	0.841
Dopamine, μg/kg/min	0.00 ± 0.00	0.02 ± 0.14	0.275	0.00 ± 0.00	0.01 ± 0.10	0.474
Norepinephrine, μg/kg/min	0.00 ± 0.00	0.00 ± 0.04	0.271	0.00 ± 0.00	0.00 ± 0.03	0.282
Dobutamine, μg/kg/min	0.00 ± 0.00	0.03 ± 0.51	0.588	0.00 ± 0.00	0.00 ± 0.00	–
At the end						
Heart rate, bpm	84.7 ± 19.6	95.6 ± 17.7	< 0.001	84.1 ± 19.8	95.5 ± 18.8	0.001
Systolic pressure, mmHg	118.4 ± 20.7	113.4 ± 16.3	0.063	117.7 ± 21.6	114.2 ± 16.7	0.297
Diastolic pressure, mmHg	61.5 ± 11.4	57.0 ± 11.0	0.010	60.6 ± 10.7	57.2 ± 11.2	0.092
Mean arterial pressure, mmHg	80.4 ± 13.8	76.1 ± 12.9	0.036	80.0 ± 13.9	76.5 ± 12.1	0.127
Central venous pressure, mmHg	9.3 ± 4.7	11.0 ± 2.7	0.001	9.6 ± 4.4	10.9 ± 2.9	0.043
Temperature, °C	36.1 ± 0.6	37.5 ± 0.5	< 0.001	36.1 ± 0.6	37.5 ± 0.6	< 0.001
Dopamine, μg/kg/min	0.00 ± 0.00	0.01 ± 0.13	0.346	0.00 ± 0.00	0.00 ± 0.00	–
Norepinephrine, μg/kg/min	0.06 ± 0.12	0.02 ± 0.05	< 0.001	0.06 ± 0.11	0.02 ± 0.04	< 0.001
Dobutamine, μg/kg/min	0.13 ± 1.02	0.29 ± 1.24	0.392	0.16 ± 1.10	0.24 ± 1.16	0.686

*mL: milliliters; bpm: beats per minute*

Data presented as mean ± standard deviation or number / total (percentage)

^a^Comparison of differences between the two groups using the *t* test for continuous variables and the χ^2^ test for categorical variables

^b^Adjusted by age, Child, MELD, presence of HCC and pre-transplantation hemoglobin

### Primary outcome

The proportion of patients receiving any transfusion of blood product components was 35.2% in the intervention phase and 56.3% in the control phase (*p* = 0.006) (Table 3). When considering the adjustment for potential confounders, patients in the intervention phase still had a lower risk of any transfusion of blood product components compared to those in the control phase (adjusted OR, 0.25; 95% CI, 0.10–0.63; *p* = 0.003) (Additional file 1: Table S1). After propensity score matching, the proportion of patients that received any transfusion of blood product components was still lower in the intervention phase (37.0 vs 58.4%; *p* = 0.018; OR, 0.42; 95% CI, 0.20–0.87; *p* = 0.019) (Table 3 and Additional file 1: Table S2).

**Table 3:** Transfusion of blood products

	Unmatched Cohort (*n* = 237)			Matched Cohort (*n* = 135)^b^		
	Intervention (*n* = 54)	Control (*n* = 183)	*p* value^a^	Intervention (*n* = 46)	Control (*n* = 89)	*p* value^a^
Transfusion of hemocomponents						
Any transfusion of hemocomponents	19 / 54 (35.2)	103 / 183 (56.3)	0.006	17 / 46 (37.0)	52 / 89 (58.4)	0.018
Red blood cells	16 / 53 (30.2)	96 / 183 (52.5)	0.004	14 / 45 (31.1)	47 / 89 (52.8)	0.017
Units transfused	0.7 ± 1.3	1.7 ± 2.7	0.007	0.6 ± 1.0	1.7 ± 2.7	0.008
Fresh frozen plasma	3 / 53 (5.7)	50 / 183 (27.3)	< 0.001	3 / 45 (6.7)	25 / 89 (28.1)	0.003
Units transfused	0.2 ± 0.8	2.1 ± 4.2	0.001	0.2 ± 0.9	2.2 ± 4.5	0.004
Cryoprecipitate	3 / 54 (5.6)	11 / 183 (6.0)	0.900	3 / 46 (6.5)	5 / 89 (5.6)	0.833
Units transfused	0.4 ± 2.1	0.4 ± 1.8	0.876	0.5 ± 2.3	0.4 ± 1.9	0.938
Platelets	10 / 54 (18.5)	31 / 183 (16.9)	0.787	10 / 46 (21.7)	16 / 89 (18.0)	0.599
Units transfused (random)	0.0 ± 0.0	0.1 ± 0.6	0.443	0.0 ± 0.0	0.1 ± 0.6	0.474
Units transfused (apheresis)	0.2 ± 0.4	0.2 ± 0.4	0.871	0.2 ± 0.4	0.2 ± 0.5	0.963
Transfusion of hemoderivatives						
Any transfusion of hemoderivatives	19 / 54 (35.2)	0 / 183 (0.0)	< 0.001	17 / 46 (37.0)	0 / 89 (0.0)	< 0.001
Fibrinogen concentrate	18 / 54 (33.3)	0 / 183 (0.0)	< 0.001	16 / 46 (34.8)	0 / 89 (0.0)	< 0.001
Grams transfused	1.4 ± 2.3	0.0 ± 0.0	< 0.001	1.4 ± 2.4	0.0 ± 0.0	< 0.001
Prothrombin complex concentrate	6 / 54 (11.1)	0 / 183 (0.0)	< 0.001	5 / 46 (10.9)	0 / 89 (0.0)	0.001
Units transfused	222.2 ± 711.5	0.0 ± 0.0	< 0.001	195.6 ± 645.3	0.0 ± 0.0	0.005
Use of antifibrinolytic	8 / 54 (14.8)	77 / 182 (42.3)	< 0.001	7 / 46 (15.2)	36 / 88 (40.9)	< 0.001
Tranexamic acid	8 / 54 (14.8)	0 / 182 (0.0)		7 / 46 (15.2)	0 / 88 (0.0)	
Aprotinin	0 / 54 (0.0)	29 / 182 (15.9)		0 / 46 (0.0)	15 / 88 (17.0)	
Epsilon-aminocaproic acid	0 / 54 (0.0)	48 / 182 (26.4)		0 / 46 (0.0)	21 / 88 (23.9)	

Data presented as mean ± standard deviation or number / total (percentage)

^a^Comparison of differences between the two groups using the *t* test for continuous variables and the χ^2^ test for categorical variables

^b^Adjusted by age, Child, MELD, presence of HCC and pre-transplantation hemoglobin

Patients in the intervention phase received less RBC (30.2 vs 52.5%; *p* = 0.004; adjusted OR, 0.21; 95% CI, 0.08–0.56; *p* = 0.002) and FFP (5.7 vs 27.3%; *p* < 0.001; adjusted OR, 0.11; 95% CI, 0.03–0.43; *p* = 0.002) (Table 3 and Additional file 1: Table S1). There was no difference regarding transfusion of cryoprecipitate and platelets.

### Secondary outcomes

Secondary outcomes are provided in Tables 3 and 4. The use of hemoderivates was higher in the intervention phase (35.2 vs 0.0%; *p* < 0.001) and the use of antifibrinolytic agents was lower (14.8 vs 42.3%; *p* < 0.001; adjusted OR, 0.33; 95% CI, 0.13–0.80; *p* = 0.015) (Table 3 and Additional file 1: Table S1). The results after the propensity score matching yielded the same results (Table 3 and Additional file 1: Table S2).

**Table 4:** Clinical outcomes after transplantation

	Unmatched Cohort (*n* = 237)			Matched Cohort (*n* = 135)^b^		
	Intervention (*n* = 54)	Control (*n* = 183)	*p* value^a^	Intervention (*n* = 46)	Control (*n* = 89)	*p* value^a^
Related to the procedure						
2003Any complication	25 / 53 (47.2)	99 / 183 (54.1)	0.373	21 / 45 (46.7)	44 / 89 (49.4)	0.761
Upper digestive hemorrhage	10 / 53 (18.9)	54 / 174 (31.0)	0.084	10 / 45 (22.2)	27 / 84 (32.1)	0.235
Arterial thrombosis	1 / 53 (1.9)	6 / 172 (3.5)	0.557	1 / 45 (2.2)	2 / 82 (2.4)	0.938
General						
Duration of mechanical ventilation	0.5 ± 1.1	1.1 ± 3.9	0.242	0.5 ± 1.2	0.9 ± 1.4	0.110
Survivors	0.4 ± 1.1	0.8 ± 1.2	0.052	0.4 ± 1.1	0.8 ± 1.4	0.094
ICU length of stay	3.2 ± 4.0	4.2 ± 6.6	0.290	3.4 ± 4.3	3.6 ± 4.6	0.781
Survivors	2.8 ± 2.7	3.6 ± 5.3	0.306	2.9 ± 2.9	3.5 ± 4.6	0.463
Hospital length of stay	12.1 ± 8.9	17.2 ± 15.4	0.022	12.4 ± 9.5	16.1 ± 16.6	0.172
Survivors	11.3 ± 7.2	16.3 ± 12.7	0.007	11.6 ± 7.5	15.1 ± 11.4	0.066
In-hospital mortality	1 / 53 (1.9)	11 / 182 (6.0)	0.226	1 / 45 (2.2)	5 / 89 (5.6)	0.369

*ICU: intensive care unit*

Data presented as mean ± standard deviation or number / total (percentage)

^a^Comparison of differences between the two groups using the t test for continuous variables and the χ2 test for categorical variables

^b^Adjusted by age, Child, MELD, presence of HCC and pre-transplantation hemoglobin

There was no difference regarding complications related to the procedure, duration of mechanical ventilation, ICU length of stay and hospital mortality among the two groups (Table 4 and Additional file 1: Table S3). However, hospital length of stay in survivors was lower in the intervention phase (11.3 ± 7.2 vs 16.3 ± 12.7 days; *p* = 0.007; adjusted β coefficient, − 5.84; 95% CI, − 9.77 – -1.91; *p* = 0.004) (Table 4 and Additional file 1: Table S3). After propensity score matching, there was only a trend toward decreased hospital length of stay in survivors in the intervention phase (11.6 ± 7.5 vs 15.1 ± 11.4 days; *p* = 0.066; adjusted β coefficient, − 3.53; 95% CI, − 7.22 – 0.17; *p* = 0.061) (Table 4 and Additional file 1: Table S4).

## Discussion

In this observational study the use of a transfusion algorithm based on ROTEM® and on the use of synthetic factor concentrates resulted in a reduction in transfusion rates of any blood product components, and in a reduction in the use of antifibrinolytic medications. No patient in the treatment group developed any major complications related to the use of the protocol.

The present study is unprecedented when introducing to liver transplantations a VET-based transfusion algorithm including the use of synthetic factor concentrates and using prospectively an earlier amplitude evaluation in the EXTEM at the fifth minute (A5_EX_), and associating it with the amplitude of FIBTEM at the tenth minute (A10_FIB_) to support transfusion therapy with either fibrinogen or platelets.

The perioperative period of liver transplantation may result in hemostatic unbalance and massive bleeding, which often leads to a treatment based on most probable deficiencies or on laboratory results that do not reflect in vivo hemostasis. Indeed, recent studies emphasize that conventional coagulation tests have significant limitations in this scenario, such as a longer time to provide useful results, absence of correlation with the risk of intraoperative bleeding, and lack of evaluation of anticoagulant factors, fibrinolysis and platelet dysfunction [25, 35–38].

This study supports the results of previous studies that showed the effectiveness of VET in the evaluation and treatment of bleeding in high complex surgeries such as cardiovascular [39], trauma [40] and liver transplantations surgeries [29, 30, 41, 42]. The use of synthetic factor concentrates (FC and PCC) in the context of coagulopathies requiring treatment, although still an off-label treatment, has been investigated previously, and a recent study showed the safety of this therapeutic option in liver transplant patients [43]. In our study we used synthetic factor concentrates predominately, as a good alternative to replace FFP and cryoprecipitate transfusions, avoiding their intrinsic complications.

FC has been shown to be effective in the treatment of patients with hypofibrinogenemia in obstetric [44], cardiac [45], and trauma surgeries [46], improving clot function and reducing bleeding. Some authors argue that in situations where cryoprecipitate is indicated, replacement with FC offers advantages from the point of view of efficacy and safety [47]. Alternatives to the treatment of hypofibrinogenemia are limited. FFP contains insufficient amounts of fibrinogen [48] and is inefficient in the clinical situations in which it is used for fibrinogen replacement [49]. Cryoprecipitate is the therapy of choice, but offers high risks of complications such as transmission of infectious diseases, acute lung injury and immuno-mediated complications, increasing morbidity and mortality in transfused patients. Besides, high contents of von-Willebrand factor, factors VIII and XIII can potentially lead to hypercoagulation in the setting of endothelial dysfunction, contributing to the development of thromboembolic events [50–52].

It is important to note that although we did not find a difference between cryoprecipitate transfusion rates in both groups, the use of VET led to an increase in the general indication of fibrinogen replacement in the intervention group, which was done with FC. One possible explanation is that the faster evaluation of coagulation when using VET directed the treatment of coagulopathy with replacement. It is known that the majority of patients undergoing liver transplantation present intraoperative hypofibrinogenemia [53, 54].

FFP remains the main therapy for multifactorial coagulopathy observed in hepatic transplantation [55–57], and PCC was initially presented as an option for reversal of coumarin anticoagulant agents [58]. Although it does not contain all the factors present in FFP, since it is composed of the vitamin K dependent factors (II, VII, IX and X) and protein C and S anticoagulant factors, the PCC contains important effectors in the coagulation, and therefore, it is an alternative in cases in which the FFP is indicated [59]. It presents a low risk of thromboembolic events, and offers the advantage of lower risk of infection transmission and transfusion reactions when compared to FFP, besides low impact on the patient’s blood volume, reducing the risk of volume overload and dilutional anemias [58, 60]. In the present study we did not find any difference in the incidence of thrombotic complications between the phases studied. A recent study showed that PCC may be more effective than FFP to restore thrombin generation in patients undergoing liver transplantation, and that the required dose is less than the dose used for warfarin reversal [61].

Transfusional triggers associated to VET are not well stablished in the scientific literature. Most of the studies that propose an algorithm based on VET use the evaluation of the amplitude in the EXTEM at the tenth minute (A10_EX_) [36, 41, 57, 62]. The use of A5_EX_ has already been shown as an effective parameter to detect thrombocytopenia and hypofibrinogenemia in patients undergoing liver transplantation [63].

Hyperfibrinolysis is an important cause of bleeding in patients undergoing liver transplantation [64]. Thus, antifibrinolytic drugs are used to reduce blood loss and transfusion of blood components, reducing costs and complications, and the decision to use this resource should be individualized because of the theoretical risk of thromboembolic events, which is still a matter of debate [65, 66]. The high incidence of fibrinolysis disturbances found in patients undergoing liver transplantation has made the use of antifibrinolytics desirable in the past, with the exclusion of patients who are more prone to thrombotic events, such as patients with inflammatory diseases of the biliary tract, previous history of thrombotic events and patients with cellular hepatocarcinoma or other types of cancer. However, it has been shown that in most situations where fibrinolysis is present in a liver transplant, it is transient and do not need intervention [67]. The introduction of ROTEM as a tool for the evaluation of coagulation allows the identification of patients who are prone to fibrinolysis and patients who are bleeding because of fibrinolysis, and these are the patients who benefit from the use of antifibrinolytics [68, 69]. Such targeted treatment may be a possible explanation of the reduction in the use of antifibrinolytics observed in our study. Finally, it is important to state that ROTEM can point to the possibility of hyperfibrinolysis if there is an increased clot lysis, but the diagnosis is possible after another specific test (APTEM) is performed, showing an improvement with the use of antifibrinolytic drugs, and this diagnosis takes time [70].

Our study has some limitations, including the small sample size, single center design, and the use of non-concurrent controls. We did not collect and include in our models patients’ characteristics regarding ICU admission and the use of preoperative mechanical ventilation, dialysis, and vasopressors. Furthermore, the intervention and control periods occurred during different periods, without blinding in the prospective group and it is not possible to control practice parameters that may have changed, for example if the surgeons became more experienced, or if the anesthesiologist had more attention to limiting blood products or a different anesthesia practice pattern. Besides, we do not have data on quantitative blood loss, there was not a standard procedure to guide transfusions in the control group and some patients in the intervention group needed cryoprecipitate after the use of FC, possibly due to lack of other coagulation factors not available in the synthetic concentrate (factors VIII, XIII or von-Willebrand).

In conclusion, our data show that the introduction of a VET-guided transfusion algorithm with the use of synthetic factor concentrates reduces the transfusion rates of allogenic blood in patients submitted to liver transplantation without increasing the risk of thrombosis. Further studies are necessary to identify whether there is an impact on the morbidity and mortality of these patients.

## Additional files

Additional file 1: Association between viscoelastic tests-guided therapy with synthetic factor concentrates and allogenic blood transfusion in liver transplantation: a before-after study – Online Supplement. These **Table S1, S2, S3, S4** contain data analysis related to transfusion of blood products and clinical outcomes in both the unmatched and matched cohorts. (DOCX 32 kb)

## Abbreviations

APTT: Activated Partial Thromboplastin Time
BIS: Bi-spectral Index
CI: Confidence Interval
CVP: Central Venous Pressure
FFP: fresh frozen plasma
HCC: Hepatocellular Carcinoma
ICU: Intensive Care Unit
INR: International Normalized Ratio
MAP: Mean Arterial Pressure
MELD: Model of End-stage Liver Disease
OR: Odds Ratio
PPC: Prothrombin Complex Concentrate
RBC: red blood cells
ROTEM: Rotational thromboelastometry
TEG: Thromboelastography
VET: Viscoelastic tests
