# Supplementary material for: Association between viscoelastic tests-guided therapy with synthetic factor concentrates and allogenic blood transfusion in liver transplantation: a before-after study
Source: BMC Anesthesiol. 2018 Dec 22;18:198. doi: 10.1186/s12871-018-0664-8 (PMC6303918; doi:10.1186/s12871-018-0664-8)
Supplement: Supplementary file 1 — Association between viscoelastic tests-guided therapy with synthetic factor concentrates and allogenic blood transfusion in liver transplantation: a before-after study – Online Supplement. These Table S1, S2, S3, S4 contain data analysis related to transfusion of blood products and clinical outcomes in both the unmatched and matched cohorts. (DOCX 32 kb) [file 12871_2018_664_MOESM1_ESM.docx]

**ASSOCIATION BETWEEN VISCOELASTIC TESTS-GUIDED THERAPY WITH SYNTHETIC FACTOR CONCENTRATES AND ALLOGENIC BLOOD TRANSFUSION IN LIVER TRANSPLANTATION: A BEFORE-AFTER STUDY**

ONLINE SUPPLEMENT

Raffael Pereira Cezar Zamper MD, Thiago Chaves Amorim MD, Veronica Neves Fialho MD, Jordana Dantas de Oliveira Lira MD, Luiz Guilherme Villares da Costa MD PhD, Flavio Takaoka MD PhD, Nicole P Juffermans MD PhD, Ary Serpa Neto MD MSc PhD

| **Table S1 – Transfusion of blood products in the unmatched cohort** | | | | | |
| --- | --- | --- | --- | --- | --- |
|  | **Unmatched Cohort (*n* = 237)** | | | **Adjusted Logistic Regression^b^** | |
|  | **Intervention**  **(*n* = 54)** | **Control**  **(*n* = 183)** | ***p* value^a^** | **Odds Ratio (95% Confidence Interval)** | ***p* value^a^** |
| **Transfusion of hemocomponents** | | | | | |
| Any transfusion of hemocomponents | 19 / 54 (35.2) | 103 / 183 (56.3) | 0.006 | 0.25 (0.10 – 0.63) | 0.003 |
| Red blood cells | 16 / 53 (30.2) | 96 / 183 (52.5) | 0.004 | 0.21 (0.08 – 0.56) | 0.002 |
| Fresh frozen plasma | 3 / 53 (5.7) | 50 / 183 (27.3) | < 0.001 | 0.11 (0.03 – 0.43) | 0.002 |
| Cryoprecipitate | 3 / 54 (5.6) | 11 / 183 (6.0) | 0.900 | 0.75 (0.15 – 3.73) | 0.722 |
| Platelets | 10 / 54 (18.5) | 31 / 183 (16.9) | 0.787 | 0.76 (0.27 – 2.12) | 0.597 |
| **Transfusion of hemoderivatives** | | | | | |
| Any transfusion of hemoderivatives | 19 / 54 (35.2) | 0 / 183 (0.0) | < 0.001 | --- | --- |
| Fibrinogen concentrate | 18 / 54 (33.3) | 0 / 183 (0.0) | < 0.001 | --- | --- |
| Prothrombin complex concentrate | 6 / 54 (11.1) | 0 / 183 (0.0) | < 0.001 | --- | --- |
| Use of antifibrinolytic | 8 / 54 (14.8) | 77 / 182 (42.3) | < 0.001 | 0.33 (0.13 – 0.80) | 0.015 |
| Data presented as number / total (percentage)  ^a^ Comparison of differences between the two groups using the χ^2^ test  ^b^ Adjusted by age, Child, MELD, presence of HCC, pre-transplantation hemoglobin, albumin, urea and creatinine. Control phase as reference | | | | | |

| **Table S2 – Transfusion of blood products in the matched cohort** | | | | | |
| --- | --- | --- | --- | --- | --- |
|  | **Matched Cohort (*n* = 135)** | | | **Logistic Regression^b^** | |
|  | **Intervention**  **(*n* = 46)** | **Control**  **(*n* = 89)** | ***p* value^a^** | **Odds Ratio (95% Confidence Interval)** | ***p* value^a^** |
| **Transfusion of hemocomponents** | | | | | |
| Any transfusion of hemocomponents | 17 / 46 (37.0) | 52 / 89 (58.4) | 0.018 | 0.42 (0.20 – 0.87) | 0.019 |
| Red blood cells | 14 / 45 (31.1) | 47 / 89 (52.8) | 0.017 | 0.40 (0.19 – 0.86) | 0.019 |
| Fresh frozen plasma | 3 / 45 (6.7) | 25 / 89 (28.1) | 0.003 | 0.18 (0.05 – 0.64) | 0.008 |
| Cryoprecipitate | 3 / 46 (6.5) | 5 / 89 (5.6) | 0.833 | 1.17 (0.27 – 5.14) | 0.833 |
| Platelets | 10 / 46 (21.7) | 16 / 89 (18.0) | 0.599 | 1.27 (0.52 – 3.07) | 0.600 |
| **Transfusion of hemoderivatives** | | | | | |
| Any transfusion of hemoderivatives | 17 / 46 (37.0) | 0 / 89 (0.0) | < 0.001 | --- | --- |
| Fibrinogen concentrate | 16 / 46 (34.8) | 0 / 89 (0.0) | < 0.001 | --- | --- |
| Prothrombin complex concentrate | 5 / 46 (10.9) | 0 / 89 (0.0) | 0.001 | --- | --- |
| Use of antifibrinolytic | 7 / 46 (15.2) | 36 / 88 (40.9) | < 0.001 | 0.26 (0.10 – 0.64) | 0.004 |
| Data presented as number / total (percentage)  ^a^ Comparison of differences between the two groups using the χ^2^ test  ^b^ After the propensity-matching. Control phase as reference | | | | | |

| **Table S3 – Clinical outcomes after transplantation in the unmatched cohort** | | | | | |
| --- | --- | --- | --- | --- | --- |
|  | **Unmatched Cohort (*n* = 237)** | | | **Adjusted Logistic/Linear Regression^b^** | |
|  | **Intervention**  **(*n* = 54)** | **Control**  **(*n* = 183)** | ***p* value^a^** | **Odds Ratio (95% Confidence Interval)** | ***p* value^a^** |
| **Related to the procedure** | | | | | |
| Any complication | 25 / 53 (47.2) | 99 / 183 (54.1) | 0.373 | 0.92 (0.46 – 1.86) | 0.817 |
| Upper digestive hemorrhage | 10 / 53 (18.9) | 54 / 174 (31.0) | 0.084 | 0.46 (0.18 – 1.17) | 0.105 |
| Arterial thrombosis | 1 / 53 (1.9) | 6 / 172 (3.5) | 0.557 | 0.73 (0.12 – 9.69) | 0.800 |
| **Transfusion of hemoderivatives** | | | | | |
| Duration of mechanical ventilation^c^  Survivors | 0.5 ± 1.1  0.4 ± 1.1 | 1.1 ± 3.9  0.8 ± 1.2 | 0.242  0.052 | -1.02 (-2.20 – 0.15)  -0.50 (-0.89 – -0.11) | 0.088  0.012 |
| ICU length of stay^c^  Survivors | 3.2 ± 4.0  2.8 ± 2.7 | 4.2 ± 6.6  3.6 ± 5.3 | 0.290  0.306 | -1.91 (-3.93 – 0.11)  -1.41 (-2.99 – 0.18) | 0.064  0.081 |
| Hospital length of stay^c^  Survivors | 12.1 ± 8.9  11.3 ± 7.2 | 17.2 ± 15.4  16.3 ± 12.7 | 0.022  0.007 | -6.06 (-10.8 – -1.34)  -5.84 (-9.77 – -1.91) | 0.012  0.004 |
| In-hospital mortality | 1 / 53 (1.9) | 11 / 182 (6.0) | 0.226 | 0.18 (0.02 – 1.71) | 0.135 |
| Data presented as mean ± standard deviation or number / total (percentage)  ^a^ Comparison of differences between the two groups using the t test for continuous variables and the χ2 test for categorical variables  ^b^ Adjusted by age, Child, MELD, presence of HCC, pre-transplantation hemoglobin, albumin, urea and creatinine. Control phase as reference  ^c^ In this models the β coefficient is reported | | | | | |

| **Table S4 – Clinical outcomes after transplantation in the matched cohort** | | | | | |
| --- | --- | --- | --- | --- | --- |
|  | **Matched Cohort (*n* = 135)** | | | **Logistic/Linear Regression^b^** | |
|  | **Intervention**  **(*n* = 46)** | **Control**  **(*n* = 89)** | ***p* value^a^** | **Odds Ratio (95% Confidence Interval)** | ***p* value^a^** |
| **Related to the procedure** | | | | | |
| Any complication | 21 / 45 (46.7) | 44 / 89 (49.4) | 0.761 | 0.89 (0.44 – 1.83) | 0.762 |
| Upper digestive hemorrhage | 10 / 45 (22.2) | 27 / 84 (32.1) | 0.235 | 0.60 (0.26 – 1.39) | 0.237 |
| Arterial thrombosis | 1 / 45 (2.2) | 2 / 82 (2.4) | 0.938 | 0.91 (0.08 – 10.31) | 0.939 |
| **Transfusion of hemoderivatives** | | | | | |
| Duration of mechanical ventilation^c^  Survivors | 0.5 ± 1.2  0.4 ± 1.1 | 0.9 ± 1.4  0.8 ± 1.4 | 0.110  0.094 | -0.39 (-0.86 – 0.08)  -0.41 (-0.89 – 0.06) | 0.104  0.088 |
| ICU length of stay^c^  Survivors | 3.4 ± 4.3  2.9 ± 2.9 | 3.6 ± 4.6  3.5 ± 4.6 | 0.781  0.463 | -0.23 (-1.85 – 1.38)  -0.57 (-2.08 – 0.94) | 0.779  0.458 |
| Hospital length of stay^c^  Survivors | 12.4 ± 9.5  11.6 ± 7.5 | 16.1 ± 16.6  15.1 ± 11.4 | 0.172  0.066 | -3.67 (-8.86 – 1.53)  -3.53 (-7.22 – 0.17) | 0.166  0.061 |
| In-hospital mortality | 1 / 45 (2.2) | 5 / 89 (5.6) | 0.369 | 0.38 (0.04 – 3.37) | 0.386 |
| Data presented as mean ± standard deviation or number / total (percentage)  ^a^ Comparison of differences between the two groups using the t test for continuous variables and the χ2 test for categorical variables  ^b^ After the propensity-matching. Control phase as reference  ^c^ In this models the β coefficient is reported | | | | | |
